# Post-traumatic stress symptom clusters in acute whiplash associated disorder and their prediction of chronic pain-related disability

**DOI:** 10.1097/PR9.0000000000000631

**Published:** 2017-11-27

**Authors:** Annick Maujean, Matthew J. Gullo, Tonny Elmose Andersen, Sophie Lykkegaard Ravn, Michele Sterling

**Affiliations:** a Recover Injury Research Centre, NHMRC Centre of Research Excellence in Road Traffic Injury Recovery, The University of Queensland, Australia; b Menzies Health Institute Qld, Griffith University, Australia; c Centre for Youth Substance Abuse Research, The University of Queensland, Australia; d Department of Psychology, University of Southern Denmark, Odense, Denmark; e The Specialized Hospital for Polio and Accident Victims, Roedovre, Denmark

**Keywords:** Whiplash, Post-traumatic stress, PTSD, Neck pain, Disability

## Abstract

**Introduction:**

The presence of post-traumatic stress disorder (PTSD) symptoms has been found to be associated with an increased risk of persisting neck pain and disability in motor vehicle crash (MVC) survivors with whiplash injuries. The findings are mixed as to which PTSD symptom(s) best predicts recovery in this population.

**Objectives:**

The aims were (1) to explore the factor structure of the Post-traumatic Stress Diagnostic Scale (PDS) in a sample of acute whiplash-injured individuals following a MVC and (2) to identify the PTSD-symptom clusters that best predict long-term neck pain-related disability in this population as measured by the Neck Pain Disability Index (NDI).

**Methods:**

A sample (N = 146) of whiplash-injured individuals completed the NDI and the PDS at baseline (<1 month) and at 6 months follow-up.

**Results:**

Principal component analyses generated 2 symptom clusters: re-experiencing/avoidance and hyperarousal/numbing. Nine trauma-related PTSD symptoms loaded exclusively on the re-experiencing/avoidance cluster and 7 nonspecific PTSD symptoms loaded exclusively on the hyperarousal/numbing cluster. One PTSD symptom (ie, inability to recall an important aspect of the trauma) had no salient loading on either clusters. Structural equation modelling analysis indicated that there was a significant positive relationship between the hyperarousal/numbing symptom cluster and long-term neck pain-related disability, while no significant relationship was found between the re-experiencing/avoidance symptom cluster and long-term neck pain-related disability.

**Conclusion:**

Given that only the hyperarousal/numbing symptom cluster predicted long-term neck pain-related disability, this finding may have implications in terms of diagnosis, assessment, and management of the psychological impact of whiplash-injured individuals following a MVC.

## Abstract 2

Two symptom clusters were generated comprising specific (intrusion/avoidance cluster) and nonspecific (hyperarousal/numbing cluster) post-traumatic stress disorder symptoms. The nonspecific post-traumatic stress disorder symptoms cluster better predicted chronic pain-related disability.

## 1. Introduction

There is evidence that symptoms of the post-traumatic stress disorder (PTSD) are prevalent in whiplash-injured individuals following a motor vehicle crash (MVC), with approximately 25% meeting the diagnostic criteria for PTSD.^4,36^ Post-traumatic stress disorder is characterised by a constellation of symptoms that can arise when a person experiences a traumatic event such as a MVC.^2^ As outlined in the Diagnostic and Statistical Manual for Mental Disorders—Fourth Edition (DSM-IV, DSM-IV TR),^1,2^ PTSD comprises 3 symptom clusters, ie, re-experiencing, avoidance, and hyperarousal. Most studies to date have used the DSM-IV to assess PTSD symptoms and identify symptom clusters likely to predict poor functional recovery in MVC survivors.

Several studies have assessed the PTSD-symptom factor structure as proposed by the DSM-IV in samples of MVC survivors with few studies supporting the DSM-IV 3-factor model and most reporting a structure comprising 2 or 4 factors. For example, Taylor et al.^40^ conducted an exploratory factor analysis and found that PTSD symptoms reported by a sample of severe MVC survivors and United Nations peacekeepers were best conceptualised using a 2-factor structure: intrusion/avoidance and hyperarousal/numbing. This 2-factor structure was replicated in another study conducted by Buckley et al.^12^ using confirmatory factor analysis (CFA) based on a sample of severe MVC survivors. Elklit and Shevlin^21^ tested 6 confirmatory factor models of the DSM-IV PTSD-symptom clusters on a large sample comprising mostly of whiplash-injured individuals following a MVC. They found that a 4-factor symptom structure was the best fitting model, namely re-experiencing, avoidance, dysphoria, and arousal. Finally, a study conducted by Beck et al.^9^ using a sample of 182 MVC survivors provided support for the DSM-IV 3 PTSD-symptom clusters.

The presence of PTSD symptoms has been found to play an important role in the persistence of whiplash symptoms.^13,27,37,41^ Several studies have shown that PTSD symptoms are predictive of later chronic neck pain and disability following a whiplash injury.^13,37,38^ Whiplash-injured individuals who experience moderate to severe pain and disability at 6 to 12 months post injury are also more likely to report more severe PTSD symptoms.^37^

Consequently, several studies have attempted to identify the PTSD-symptom clusters likely to predict poor recovery in this population. Earlier research using the DSM-III PTSD diagnostic system comprising only 2 symptom clusters, ie, re-experiencing and avoidance found that both clusters were associated with persistent whiplash complaints at 4 weeks^17^ and 6 months following a MVC.^39^ However, more recent studies using the DSM-IV 3-symptom clusters have found that hyperarousal symptoms are better predictors of poor functional recovery in this population.^13,31^ Given the ambiguous findings regarding the number of PTSD clusters and symptoms likely to predict poor recovery in whiplash-injured individuals following a MVC, the aims of the current study were 2-fold: (1) to assess the structural model of PTSD-symptom clusters using a sample of nonhospitalised whiplash-injured individuals who presented with a range of PTSD symptoms following a MVC and (2) to identify the symptom clusters that best predict long-term neck pain-related disability in this population.

## 2. Methods

### 2.1. Participants

One hundred forty-six individuals with acute whiplash were recruited within 1 month of injury via hospital emergency departments, through advertisement in the general media, and via referrals from physiotherapy practices and general practitioners in and around Brisbane, Australia. They were eligible if they had neck pain as a result of a MVC that occurred in the last 4 weeks and met the Quebec Task Force Classification of WAD I, II, or III.^34^ In addition, participants needed to be aged between 18 and 65 years and fluent in English. Participants were excluded if they were WAD IV (fracture or dislocation of the cervical spine), experienced concussion or head injury as a result of the accident, and if they reported a previous history of whiplash, neck pain, or headaches that required treatment. They were also excluded if they reported being diagnosed with or receiving treatment for a psychiatric or psychological condition either currently or in the past.

Participants were assessed at baseline (<1 month post MVC) and again at 6 months post injury. A small fee to cover travel costs associated with attending the assessment sessions was provided to the participants. Ethics approval was granted by the institutional medical research ethics committees.

### 2.2. Measures

#### 2.2.1. Post-traumatic Stress Diagnostic Scale (PDS)

Post-traumatic stress symptoms were assessed by the PDS,^23^ which is a 17-item self-report measure with each item corresponding to one of the DSM-IV PTSD symptoms. Respondents rate the frequency of each of the 17 items that reflect DSM-IV criteria “B” re-experiencing, “C” avoidance, and “D” hyperarousal on a 4-point Likert scale (0 = “not at all or only one time” to 3 = “five or more times a week/almost always”). Participants are asked to rate the frequency of symptom related to their MVC for the past 30 days. The PDS has good psychometric properties including strong internal consistency (α = 0.92) and good test–retest reliability (*r* = 0.87).^22^ The PDS also has high convergent validity with the interview version of that scale.^22,23^ Internal consistency in the current study was high (α = 0.92).

#### 2.2.2. Neck Disability Index (NDI)

The NDI is a self-report measure designed to assess neck pain–related disability and comprises 10 items pertaining to functional activities, pain intensity, concentration, and headache.^42^ These items are rated on a Likert scale from 0 (no disability) to 5 (total disability). The overall score (out of 100) is calculated by totalling the responses of each individual item and multiplying it by 2, with higher scores indicating greater levels of pain-related disability. The NDI has been shown to have good internal consistency, test-retest reliability, and validity.^42^ Cronbach α in the current study was high (α = 0.91).

### 2.3. Statistical analyses

A CFA and a principal component analysis (PCA) were used to explore the factor structure of the PDS using the Statistical Package for Social Sciences.^35^ Structural equation modelling (SEM) using the AMOS 23.0 program^5^ was used to identify the symptom clusters that best predict long-term neck disability. The model was tested using maximum likelihood estimation. In accordance with Hu and Bentler,^10,26^ the χ^2^ test was used as a statistical test of model fit (α = 0.05). This test can be overly sensitive in large samples, so the “normed” χ^2^ was also examined. This statistic is calculated as χ^2^ divided by the model degrees of freedom (ie, χ^2^/*df* χ^2^). Values of χ^2^/*df* between 1.00 and 2.00 indicate good fit, and values between 2.00 and 3.00 indicate acceptable fit.^14^ The comparative fit index (CFI) and root mean square error of approximation (RMSEA) were also used.^10^ As a general guide, Hu and Bentler^26^ suggested the following cutoffs for “acceptable” fit: CFI ≥0.90; RMSEA ≤0.10, and these were adopted for the present study. Cutoffs for “good fit” were CFI ≥0.95; RMSEA ≤0.06. However, such values should be regarded as guidelines rather than strict cutoffs when assessing a model fit.^29^

## 3. Results

### 3.1. Sample

As shown in Table 1, the sample consisted of 146 participants, 94 women and 52 men ranging in age from 18 to 65 years (mean 37.4 ± 13.7 years).

**Table 1:**
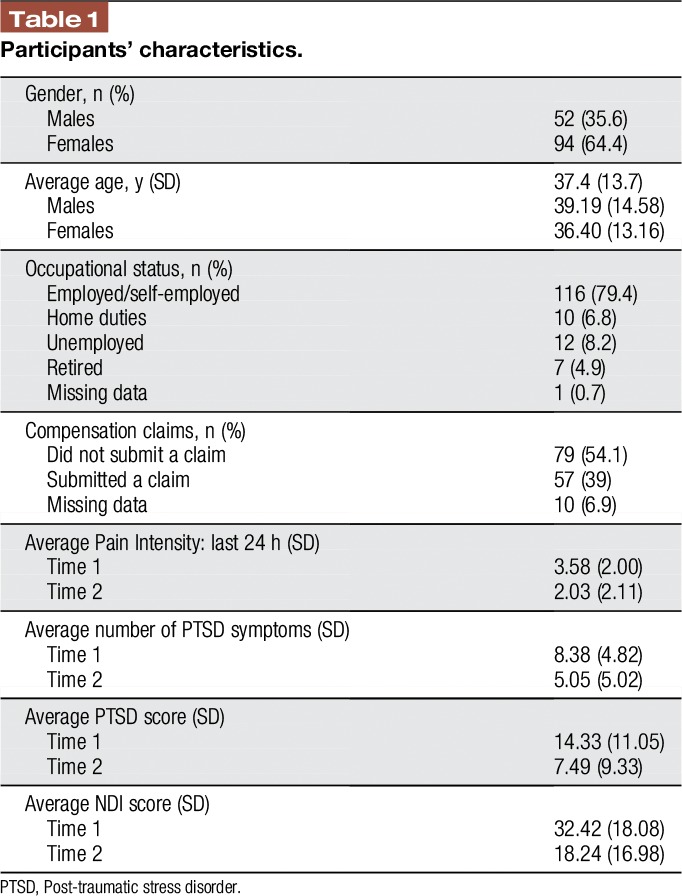
Participants' characteristics.

At entry to the study, 80% (n = 116) of the participants were employed or self-employed, and 39% (n = 57) had submitted a compensation claim. The average number of PTSD symptoms at time 1 was 8.38 (SD = 4.82), and the mean total PDS symptoms score was 14.33 (SD = 11.05). The average PDS symptoms score for this sample is similar to those previously reported in individuals with WAD^18,36^ and in a sample of MVA survivors.^19^ In addition, 70% of the participants in the current study scored below the recommended cutoff score of 18 on the PDS.^19^

Twenty-five participants (17.1%) were lost to follow-up at time 2, and these participants did not complete the PDS and NDI at time 2. The percentage of missing data for all remaining items was below 5%. The missing data were imputed using full-maximum likelihood estimation, an optimal approach for handling missing data.^25^

Independent sample *t* tests were conducted to compare the NDI and PDS scores at the 2 time points for compensation claimants and nonclaimants. There was a significant difference in NDI scores at time 1 for claimants (mean = 38.63, SD = 17.51) and nonclaimants (mean = 28.53, SD = 16.77); *t*(136) = −3.44, *P* = 0.001 and also at time 2 (claimants: mean = 23.63, SD = 17.49; nonclaimants: mean = 14.96, SD = 16.23); *t*(113) = −2.74, *P* = 0.007.

There was no significant difference in PDS scores at time 1 for claimants (mean = 15.11, SD = 11.56) and non-claimants (mean = 13.88, SD = 10.92); *t*(128) = −0.62, *P* = 0.538 nor at time 2 (claimants: mean = 9.75, SD = 10.28; nonclaimants: mean = 6.28, SD = 8.59); *t*(111) = −1.95, *P* = 0.053.

### 3.2. Factor structure of the Post-traumatic Stress Diagnostic Scale

A CFA was conducted specifying the original DSM-IV 3-factor structure (re-experiencing, avoidance, and hyperarousal). This model was found to provide poor fit to the data, χ^2^(116) = 259.62, *P* < 0.001, CFI = 0.88, RMSEA = 0.09, AIC = 367.62. Also of note, there was a very strong correlation between latent avoidance and hyperarousal (*r* = 0.87), suggesting that these may not be distinct factors. Based on these findings, a PCA was conducted to explore the factor structure within this population.

Prior to performing a PCA, the suitability of data for factor analysis were assessed. Inspection of the correlation matrix revealed the presence of many coefficients of 0.3 and above. A Kaiser–Meyer–Olkin score of 0.60 is considered necessary to reliably use factor analysis for data analysis, and scores above 0.80 are considered very good. The Kaiser–Meyer–Oklin value in the current study was 0.90, and Bartlett test of sphericity reached statistical significance, *P* < 0.001, supporting the factorability of the correlation matrix.

An initial PCA with oblimin rotation revealed the presence of 3 components with eigen values exceeding 1 (eigen values 7.62; 1.48; 1.02), explaining 59.54% of variance. Nine items loaded strongly on component 1, 7 items on component 2, and 1 item (C3: inability to recall important aspect of the trauma) loaded on component 3. Given that only 1 item loaded strongly on component 3, further investigation was carried out to decide whether item C3 should be excluded. First, we examined the association between item C3 and NDI score, and there was no significant correlation between these 2 variables at time 1 (*r* = 0.13, *P* = 0.11) nor at time 2 (*r* = 0.04, *P* = 0.65). We also checked whether the internal consistency of each component would increase with the addition of item C3. For component 1, the coefficient α was 0.897 and when item C3 was included, the coefficient α level decreased to 0.887. Similarly, for component 2, the coefficient α was 0.862 and when item C3 was included, the coefficient α level decreased to 0.845. In other words, including item C3 did not have a significant impact on the internal consistency of these 2 components. Based on these analyses, it was decided to exclude item C3 from further investigation.

A PCA with oblimin rotation was conducted on the remaining 16 items and it generated 2 components with eigen values exceeding 1 (eigen values = 7.49 and 1.48), accounting for 56.07% of variance. As shown in Table 2, all 16 items loaded exclusively on their respective component with 9 items loading on component 1 and 7 items loading on component 2. Component 1 comprises all 5 re-experiencing symptoms (B1–B5), 2 avoidance symptoms (C1 and C2), and 2 symptoms from the hyperarousal cluster (D4 and D5). Component 2 consists of 4 avoidance and 3 hyperarousal symptoms. Consistent with the study by Taylor et al.,^40^ component 1 was labelled “Intrusion/Avoidance” and component 2 “Hyperarousal/Numbing.”

**Table 2:**
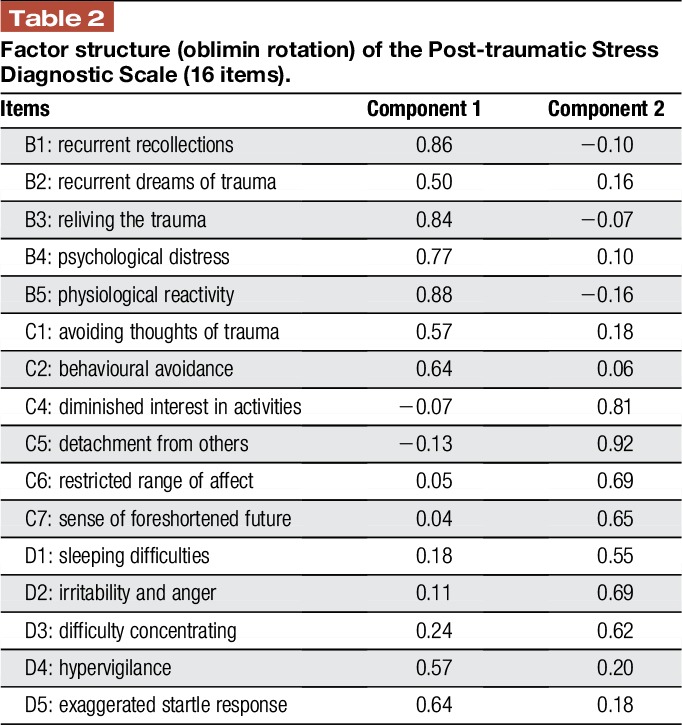
Factor structure (oblimin rotation) of the Post-traumatic Stress Diagnostic Scale (16 items).

### 3.3. Predictors of neck pain-related disability

Structural equation modelling was used to test the relative contribution of the 2 extracted components in predicting long-term neck pain-related disability. While baseline NDI scores were normally distributed, long-term NDI scores (6 months) were significantly skewed (*z*_skewness_ = 3.81, *P* < 0.001) and corrected with a square-root transformation. The hypothesized model specified a latent intrusion/avoidance factor with the 9 items loading on component 1 as indicators, and a latent hyperarousal/numbing factor with the 7 items loading on component 2 as indicators (Table 2). Both intrusion/avoidance and hyperarousal/numbing factors were hypothesized to predict long-term (6 months) neck pain–related disability, controlling for baseline disability. The latent factors were allowed to covary with each other and baseline neck pain–related disability.

**Figure 1.**
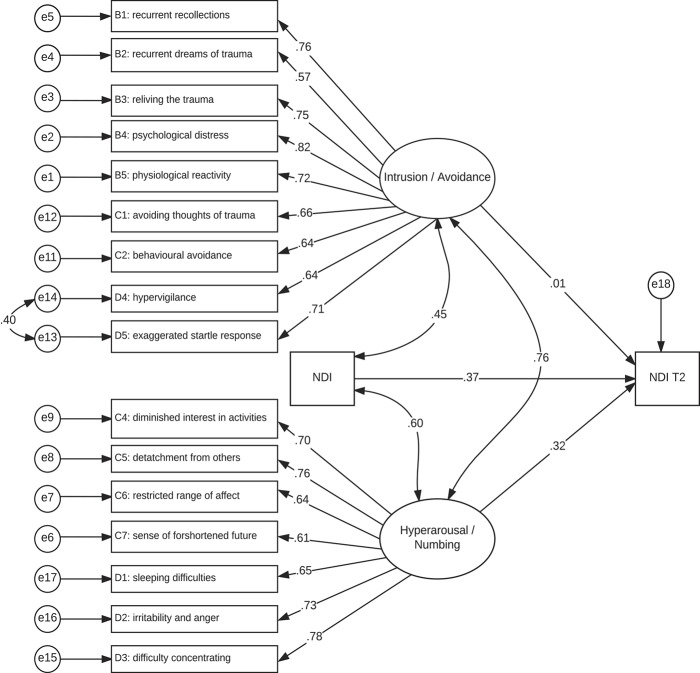
Post-traumatic stress disorder symptom clusters predicting chronic pain-related disability.

The hypothesized model was found to provide marginally acceptable fit, χ^2^(131) = 268.57, *P* < 0.001, CFI = 0.89, RMSEA = 0.09. Examination of modification indices suggested that a residual covariance between items D4: hypervigilance and D5: exaggerated startle response would enhance model fit, and this was specified. The revised model showed acceptable fit and was retained, χ^2^(130) = 247.35, *P* < 0.001, CFI = 0.91, RMSEA = 0.08. While both intrusion/avoidance and hyperarousal/numbing were associated with concurrent (baseline) neck pain–related disability, only hyperarousal/numbing significantly predicted future neck pain–related disability (unstandardized coefficient = 1.15, SE = 0.57, *P* = 0.043). In total, the model accounts for 39% of the variance in long-term neck pain–related disability. The final model is depicted in Figure 1.

Bentler^10^ recommends that SEM involving smaller samples should additionally report the test of an a priori model that is expected to be rejected (ie, have poor fit to the data). This is used to determine whether there is adequate statistical power to reject a poor-fitting model. This poor-fit model was similar to the hypothesized model but removed the covariances between predictors and also removed the direct path from hyperarousal/numbing to neck pain–related disability. As expected, this model was found to provide a poor fit to the data, χ^2^(135) = 416.33, *P* < 0.001, CFI = 0.78, RMSEA = 0.12.

## 4. Discussion

This study first examined the structural model of PTSD-symptom clusters in a sample of nonhospitalised whiplash-injured individuals who presented with a range of PTSD symptoms following a MVC. The second aim was to identify the symptom clusters that were likely to predict long-term neck pain-related disability in this population.

The current findings suggest that suspected MVC-related PTSD symptoms reported by patients with WAD are best represented by a 2-factor model distinguishing between intrusion/avoidance and hyperarousal/numbing PTSD symptoms. These results are similar to the findings of Taylor et al.^40^ and Buckley et al.^12^ who also found that a 2-factor structure was a better fit for this population than the 3-factor model proposed by the DSM-IV. Consistent with the findings of Taylor et al.^40^ and Buckley et al.,^12^ symptoms specific to PTSD such as recurrent recollections of the trauma, reliving the trauma, and avoiding thoughts of the trauma loaded saliently on the intrusion/avoidance component. By contrast, symptoms that are also commonly reported in association with whiplash pain and related disability (eg, irritability, sleeping difficulties, and decrease in activities) loaded saliently on the hyperarousal/numbing component.

In addition, the initial PCA revealed that one of the 17 PTSD symptoms, namely “recalling important aspects of the trauma” loaded exclusively and on its own on a third component, indicating that this specific symptom was measuring a different underlying construct than the other 16 PTSD symptoms and was removed from further analysis. Interestingly, Taylor et al.^40^ found that this symptom generated the weakest factor loading (<0.30), also indicating that this item measures a different underlying construct.

After the 2 symptom clusters were identified, the next step was to assess their relative contribution in predicting long-term neck pain-related disability. The SEM revealed that the hyperarousal/numbing symptoms cluster significantly predicted future neck pain–related disability, while the intrusion/avoidance cluster did not. Interestingly, the hyperarousal/numbing cluster consists of symptoms that have been identified in the literature as being nonspecific PTSD symptoms (eg, difficulty concentrating, diminished interest in activities, a sense of foreshortened future) as they overlap with symptoms from other mood and anxiety disorders.^6,20^ The inclusion of these nonspecific symptoms in the PTSD clusters has been debated^16,33,44^ because of their comorbidities with other mental health conditions. Furthermore, a valid PTSD diagnosis must be precipitated by a traumatic event.

Several studies have demonstrated that even in the absence of trauma, individuals can still present with a range of PTSD symptoms.^11,24,28,30^ One of those studies was conducted by Bodkin et al.^11^ who found that individuals who had a primary diagnosis of depression exhibited significant levels of PTSD in the absence of a traumatic event. Similarly, Mol et al.^30^ found no significant differences in levels of PTSD symptoms when comparing adults who reported experiencing a severe trauma as defined by the DSM-IV and those who did not.

In terms of the presence of PTSD symptoms in individuals with WAD following a MVC, it is important to note that PTSD and WAD do share a range of overlapping symptoms such as sleeping difficulties, irritability, and decrease interest in activities.^13^ Several theoretical models have attempted to explain the reciprocal relationship between PTSD and pain associated with WAD including the mutual maintenance model.^32^ This model proposes that various symptoms from the 3 DSM-IV PTSD clusters (re-experiencing, avoidance, and hyperarousal) maintain and intensify symptoms of pain and vice versa.^32^ The shared vulnerability model^7,8^ proposes that individual factors such as heightened sensitivity to anxiety and lower threshold to pain sensitivity predispose some individuals to develop both PTSD and chronic pain. According to Asmundson and Katz,^8^ PTSD and chronic pain are more likely to co-develop when vulnerable people (eg, high sensitivity to anxiety) are exposed to an event that is both traumatic and painful, at which point reminders of the trauma and pain sensation can elicit further alarm reactions.

When a MVC is perceived as being traumatic, these theoretical models certainly help explain the reciprocal relationship between PTSD and pain. However, when the nonspecific PTSD symptoms are present in the absence of perceived trauma, these symptoms cannot be associated with PTSD, given that there is no traumatic event. Although we cannot determine from our study if the patients perceived the MVC as traumatic, our findings that only nonspecific symptoms (hyperarousal/numbing cluster) predicted ongoing disability suggest that these symptoms may be more related to the pain and disability of WAD than the traumatic event itself.

The pain associated with whiplash injuries can detrimentally have an impact on various aspects of an individual's life. For example, many individuals decrease their work and daily activities because of pain. This sudden decrease in daily and work-related activities can generate a whole range of other issues that are associated with the aftermath of the injury such as financial problems due to reduction in work hours, which can lead to some experiencing a sense of foreshortened future. Further, the pain associated with whiplash injury can lead to an increase in sleeping difficulties and irritability. Therefore these symptoms although part of the PTSD-symptom clusters can also be triggered by issues other than MVC-related trauma, specifically issues which are associated with pain and related disability of the injury. This suggests that it is important to be perhaps cautious when using and interpreting these questionnaires such that the condition is not over “psychologised” as these symptoms may at times simply be a “normal” psychological response to the physical injury.^15^

Given from our findings that nonspecific PTSD symptoms (eg, diminished interest in activities, sense of foreshortened future, difficulties sleeping, and/or concentrating) predicted 6-month pain-related disability while specific PTSD symptoms did not, this suggests that factors related to WAD pain and disability as opposed to PTSD per se may be more important in this condition. Consequently, caution needs to be exercised when assessing PTSD symptoms in nonhospitalised whiplash-injured individuals following a MVC.

In terms of PTSD diagnosis, one of the key changes in the new DSM-5^3^ is the renewed emphasis on avoidance symptoms. Avoidance behaviours associated with trauma are key features of PTSD.^45^ In line with the DSM-5 criteria, an individual needs to present with at least 1 avoidance symptom (ie, trauma-related thoughts and feelings and/or external reminders associated with the trauma) to meet PTSD diagnostic criteria. Whereas previously in the DSM-IV and DSM-IV-TR, an individual could fulfil the criteria for the avoidance cluster (ie, Cluster C) only by endorsing 3 numbing symptoms and no avoidance symptoms. Numbing symptoms are known to be subjective general distress symptoms and nonspecific to PTSD.^33,43,46^ Consequently, an individual could meet the criteria for the avoidance cluster in the absence of perceived trauma. The DSM-5 new requirement that at least 1 avoidance symptom needs to be present is therefore a critical step in improving the accuracy of PTSD diagnosis in nonhospitalised whiplash-injured individuals following a MVC.

The present findings may have important implications in terms of diagnosis, assessment, and management of the psychological impact on whiplash-injured individuals following a MVC. Individuals who did not perceive their MVC as being a traumatic event but still present with a range of these nonspecific PTSD symptoms may not benefit from a psychological trauma intervention (eg, exposure therapy). However, they may perhaps benefit more from a psychological intervention that is predominantly based on self-management techniques to help them develop their skills and confidence in managing their recovery.

A number of limitations should be considered when interpreting the findings of this study. First, a relatively small sample was used, which limits the generalizability of the findings. A second limitation was that the CFA, the principal component analysis, and the SEM were conducted on the same group of participants; a replication utilising the split-sample strategy for cross-validation of the findings would strengthen the current findings. Finally, the analyses were conducted using the 17 symptoms listed in the DSM-IV, future research is needed to determine whether the current findings can be replicated using the 20 PTSD symptoms listed in the DSM-5.

## 5. Conclusions

The results of the current study support a 2-factor symptom clusters (intrusion/avoidance and hyperarousal/numbing). The hyperarousal/numbing cluster comprising of nonspecific PTSD symptoms was the only cluster which predicted long-term neck pain-related disability. This finding indicates that although nonhospitalised whiplash-injured individuals may present with a range of PTSD symptoms following a MVC, these symptoms can be related to the pain and disability of WAD rather than being symptoms of PTSD per se.

## Disclosures

The authors have no conflict of interest to declare.

This study was supported by an Australian Research Council Discovery Grant, Canberra, ACT.
